# Wallerian demyelination: chronicle of a cellular cataclysm

**DOI:** 10.1007/s00018-017-2565-2

**Published:** 2017-06-09

**Authors:** Nicolas Tricaud, Hwan Tae Park

**Affiliations:** 1 INSERM U1051, Institut des Neurosciences de Montpellier (INM), Université de Montpellier, Montpellier, France; 2 Peripheral Neuropathy Research Center, Department of Physiology, College of Medicine, Dong-A University, Busan, South Korea

**Keywords:** Myelin, Degeneration, Peripheral nervous system, Schwann cells, Wallerian demyelination, Cellular reprogramming

## Abstract

Wallerian demyelination is characteristic of peripheral nerve degeneration after traumatic injury. After axonal degeneration, the myelinated Schwann cell undergoes a stereotypical cellular program that results in the disintegration of the myelin sheath, a process termed demyelination. In this review, we chronologically describe this program starting from the late and visible features of myelin destruction and going backward to the initial molecular steps that trigger the nuclear reprogramming few hours after injury. Wallerian demyelination is a wonderful model for myelin degeneration occurring in the diverse forms of demyelinating peripheral neuropathies that plague human beings.

## Introduction

Demyelination is a tricky word to pronounce (phonetic: ) but it is nevertheless critical in human health. In the peripheral nervous system, myelin made by Schwann cells covers the majority of the axons and the loss of this myelin is termed peripheral demyelination. For clinicians, demyelination merely means the status of the nerve when myelin is gone but for biologists this also means the process through which Schwann cells lose their myelin (demyelinating cells). Indeed peripheral demyelination does not result from Schwann cells death but from a dedifferentiation process that transforms a myelinated Schwann cell into a demyelinated Schwann cell. This last cell is able to remyelinate during nerve regeneration.

The causes of peripheral demyelination are multiple: toxic (tellurium or diphtheric for example), metabolic (diabetes), infectious (*Mycobacterium leprae*), hereditary (Charcot–Marie–Tooth diseases), immune (Guillain–Barré syndrome, CIDP), thermic (hot or cold burn), ischemic, or traumatic (nerve compression, crush, or cut). Traumatic nerve injury induces Wallerian degeneration, which includes axonal degeneration and the subsequent Wallerian demyelination. Wallerian demyelination is the most common cause of peripheral nerve demyelination and probably everyone in this world has suffered this at least once. Indeed the smallest cut may easily sever a nerve bundle in the skin leading to pain, axonal degeneration, and Schwann cells demyelination. In peripheral nerves, neuronal plasticity allows axons to grow back and the nerve bundle to regenerate [1]. However, elimination of myelin is critical as myelin proteins are known to inhibit axonal regeneration [1]. Finally, demyelinated Schwann cells then remyelinate axons to restore the full nerve function.

Because nerve cut or crush induces a synchronized demyelination of Schwann cells downstream of the injury in the nerve, Wallerian demyelination has been the model of choice to study molecular and cellular mechanisms of demyelination. Recent cell biology investigations revealed that Wallerian demyelination is a stereotyped succession of molecular and cellular steps that lead to myelin degradation and loss. Microscopically visible events such as formation of myelin ovoids (small cellular chambers containing myelin clump) are typical of the late stages of Schwann cell demyelination and they were characterized using light and electron microscopy early in the twentieth century. However, these visible events result from early invisible molecular processes that occur in Schwann cell. These invisible processes were characterized only recently using new imaging, genetic, molecular, and cellular technologies.

Our goal here is to review both late cellular and early molecular processes that lead to Wallerian demyelination in Schwann cells. We propose to start with late events and to go back in time toward the initial event(s) that triggers demyelination following axonal degeneration.

## Wallerian demyelination

As originally described by Waller in 1850, degeneration of peripheral nerves after injury is announced by the production of numerous small chambers along the distal stump of peripheral nerves [2]. These drastic changes of myelin morphology are accompanied by the biochemical destruction of the myelin sheath, which appears prominently 1 week after injury. Indeed biochemical signs of demyelination such as myelin protein digestion, drop of lipid content, and cholesterol ester sharply increase after 1 week [3–6]. Before these late degenerative events are observed by biochemical and histochemical analyses, morphological changes in the myelin sheath can be detected during the first week post-injury and they can be conveniently divided into an early stage (up to 3 day) and a later stage (3–6 days) based on two pronounced characteristic features: complete axonal degeneration and macrophage infiltration. During the early stage, Schwann cell response to nerve injury is obvious as myelin ovoids appear in the cytoplasm and paranodal loops retract on the degenerating axon [7–9]. Molecularly, Schwann cells are also dedifferentiating into an immature state [10]. During the later stage (3–6 days), the number of macrophages sharply increases and these cells also start to digest myelin fragments expulsed by demyelinating Schwann cells [11, 12]. Actually since myelin ovoids retain obvious myelin structures under electron microscopy and because chemical destruction of the myelin has barely started by 1 week after injury, myelin disintegration (or breakdown) has been considered to be better terminology to describe the Schwann cell demyelination process that generates myelin ovoid [6, 12–14]. However alongside myelin ovoid formation, it has recently been suggested that myelin digestion by Schwann cells themselves also plays an important role in the biochemical destruction of myelin sheath early during Wallerian demyelination [15–18]. Thus, Schwann cell demyelination may encompass both myelin disintegration and digestion even though the respective role of each process has never been formally demonstrated.

Recent studies have demonstrated that this degenerative process of demyelination by Schwann cells is not limited to Wallerian degeneration. In hereditary demyelinating neuropathy, Schwann cell phenotype changes very much as in Wallerian degeneration [19]. In addition, demyelination induced by toxic material or immune attacks in inflammatory neuropathies also shows paranodal retraction, myelin clump—which is actually not ovoid in the respect of intact axon—and dedifferentiation indicating that these features also contribute to pathologic demyelination in various neuropathic diseases [20–23]. Thus, the understanding of mechanistic aspects of Schwann cell demyelination in Wallerian degeneration might provide an important insight into the pathognomonic mechanism of demyelinating neuropathies.

## 24 h to 3 days: myelin collapse

During the early period of Wallerian degeneration, the generation of myelin ovoids is theoretically helpful for the clearance of compact myelin sheath within Schwann cells. Since Young described the myelin fragmentation process as the result of surface tension generated by myelin itself [24], there was not much progress in the understanding of the myelin ovoid formation during Wallerian degeneration. The active involvement of Schwann cell in the generation of these small chambers was first seen as a “contraction” of reactive and hypertrophic cells [13]. Successive morphological analysis using electron microscopy by Webster [9] and Ghabriel and Allt [25] showed the stereotypic segmentation of myelin at Schmidt–Lanterman incisures. These incisures are cellular channel of cytoplasm, crossing the compact myelin in order to “irrigate” the cell [26]. Incisures collapse occurred with an increase of cytoplasm in existing incisures, incisures dilatation, and not with de novo generation of new incisures. The apparent increased number of incisures in injured nerves probably results from small uncomplete incisures being more visible [7, 25, 27, 28]. Since adherens junctions and junctional proteins such as E-cadherin present in incisures are destroyed during Wallerian degeneration [18, 29], it is unlikely that more incisures form de novo at that time. Instead, the junctional destruction may allow an increase of cytoplasm in incisures that would make them more visible in demyelinating conditions. In this sense, junctional destruction in incisures may be a first step in the initiation of myelin fragmentation. The dilatation of incisures is not limited to Wallerian degeneration as it is reported in many other demyelinating conditions including the segmental demyelination of inflammatory neuropathies [30–32]. Furthermore, duplication of peripheral myelin protein 22 gene responsible for hereditary peripheral neuropathy Charcot–Marie–Tooth disease 1A resulted in an abnormal actin structure in incisures [33, 34]. Thus, the alteration of incisural structure may be a general pathognomonic feature of demyelinating neuropathies in the nerve.

The junctional complex composed of E-cadherin/catenins is highly localized to outer mesaxon, Schmidt–Lanterman incisures, and paranodal area. Its molecular composition is very similar to epithelial adherens junctions [35, 36]. The maintenance of compact myelin structure in adult nerves requires not only myelin proteins but also appropriate localization of this junctional complex in non-compact regions including incisures [36]. There seems to be a specific mechanism by which these junctional structures in non-compact areas are dismantled during Wallerian degeneration. First of all, the selective destruction of E-cadherin is dependent on actin polymerization [18]. In normal nerves, F-actin is highly enriched in incisures [37] and new actin polymerization occurs within these structures shortly after nerve injury. The inhibition of the actin polymerization not only prevents E-cadherin destruction but also myelin fragmentation. This is actually the earliest event that relates molecular changes occurring in Schwann cells to myelin fragmentation after nerve injury. Interestingly, although the protein level of β-catenin, an intracellular component of adherens junction, is not significantly reduced during Wallerian degeneration (unpublished observation), β-catenin is released from dissolved junctional region and translocated into the Schwann cell nucleus in an actin polymerization-dependent manner, illustrating the dissolution of junctional structures [18]. The role of nuclear translocation of β-catenin in Schwann cells after injury has not been determined yet.

**Fig. 1:**
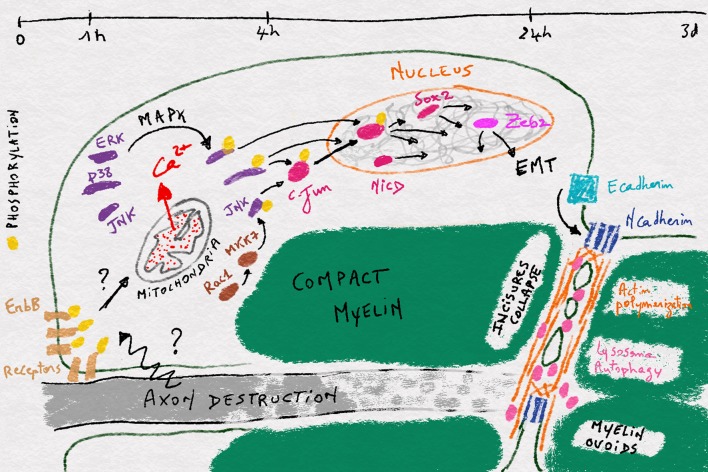
Illustration of the different steps occuring in a myelinated Schwann cell during Wallerian demyelination. The timeframe is shown at the top and between each timepoint the different events occuring in the cell during the respective time period are shown

However, actin polymerization-dependent junctional destruction does not seem to be sufficient to complete myelin fragmentation during Wallerian degeneration. Indeed the disappearance of the axon during Wallerian degeneration allows the apposition and the fusion of the innermost plasma membrane at incisures (Fig. 1). The resulting transverse cleavage requires simultaneous plasma membrane severance and repair for closing myelin around ovoids [25, 26]. This membrane remodeling seems to require a function of lysosomal enzymes which are recently considered to be essential components for membrane repair in various cellular phenomena [38–40]. Holtzman and collaborators showed the activation of lysosome in Schwann cells after injury [41]. In accordance with this, the expression of Lysosomal Associated Membrane Protein-1 (LAMP-1), a lysosomal marker, is dramatically increased in injured nerves and initially localize to incisures during myelin fragmentation [21, 42]. Morphological analyses showed that myelin membrane cleavage is not complete in peri-incisural areas when lysosome was inhibited [21] indicating that myelin fragmentation into small chamber, that definitely employs membrane cleavage/repair processes, requires lysosomal activity (Fig. 1).

Interestingly, it was recently shown that lysosomal function is actually related to an autophagy process in Wallerian demyelination (Fig. 1) [15, 21]. Autophagy is a self-eating process for recycling cellular organelles including mitochondria and peroxisome during diverse cellular events. Autophagy initiates with the formation of an isolation membrane that extends around the target organelle to form an autophagosome, which finally fuse with lysosome for the degradation of the target [43]. After peripheral nerve injury, autophagy is activated within a day in Schwann cells and mice defective for autophagy in these cells specifically exhibited delayed biochemical myelin destruction and stable compact myelin sheath even 7 days after injury [15, 21]. This is in line with the role of lysosome in myelin fragmentation. In addition, autophagy and lysosome activation have also been shown to occur in peripheral nerves during inflammatory demyelinating neuropathy, tellurium-induced neuropathy, and hereditary demyelinating neuropathy [15, 21, 44, 45]. So it is very likely that autophagy and lysosome activation is a common mechanism for Schwann cells to demyelinate in various type of demyelinating neuropathies.

## 4–24 h: cellular reprogramming and dedifferentiation

Before myelin collapse, 24 h after the initiation of Wallerian demyelination, very little changes are observed in the myelin sheath, but a complete revolution has nevertheless been engaged in the cell. Indeed demyelination is not a mere degenerative process resulting in apoptotic or necrotic cell death, but it represents a deep reprogramming of the myelinated Schwann cell [46]. Indeed the cell dedifferentiates and re-expresses genes that were active in the pro-myelinating cell or in the previous immature states [47–49]. However, while the demyelinating Schwann cell is morphologically and biochemically close to a pro-myelinating cell [10, 50], it is not just a phenocopy: Schwann cell also expresses during demyelination genes that were not expressed previously such as Sonic Hedgehog (Shh) and Olig1 [51, 52].

This step of cellular reprogramming, which starts in the nucleus around 4 h after nerve injury, is a key point to understand demyelination but it remains largely uncharted. The best characterized event in this step is the activation of the leucine-zipper zinc-finger transcription factor, c-Jun (Fig. 1). This factor is a key component of the AP-1 transcription complex and the terminal effector of the Jun-N-terminal kinase (JNK) pathway (e.g., [53]). During Wallerian demyelination, c-Jun is strongly upregulated as early as 4 h after nerve injury with a maximum activation at 12 h [54, 55]. Reducing c-Jun expression in Schwann cells significantly delays demyelination and impairs functional recovery after nerve injury [51, 55]. This transcription factor appears as a key factor of the Schwann cell reprogramming as it upregulates genes related to neuronal growth and regeneration such as N-cadherin, p75NTR, and NCAM, and the signaling molecules GDNF, artemin, Shh, and BDNF, and it downregulates myelin-related genes expression such as Egr2, Mpz, Mbp, periaxin, and E-cadherin [51, 55, 56]. C-Jun targets also include genes involved in the morphogenetic and the myelin clearing processes observed during demyelination [51, 57]. In particular, JNK-c-Jun pathway stimulates the injury-induced autophagic flux in Schwann cells [15].

Another trigger for cell reprogramming is probably the Notch pathway and its nuclear component NICD, which is also activated during Wallerian demyelination (Fig. 1). If the stimulation of this pathway is sufficient to induce demyelination in vivo [58], the molecular mechanisms that activates the pathway and mediate its effect on the cell genome remain unknown.

Beyond transcriptional changes induced by c-Jun and Notch activation several additional modifications occur in the nucleus during demyelination. Sox2, one of the group of transcription factors that induce pluripotent stem cells from adult somatic cells [59], is strongly upregulated in Schwann cells during Wallerian demyelination [60], suggesting an increased chromatin plasticity. Interestingly Sox2 remains normally upregulated in injured nerves of mice deleted for c-Jun indicating it is not genetically downstream of c-Jun [55]. So an additional mechanism may activate Sox2 expression and transcriptional plasticity in demyelinating cells (Fig. 1). Histone deacetylase HDAC2 is also upregulated 24 h after nerve injury in mouse sciatic nerve [61]. This epigenetic factor promotes the expression of Oct6, a key pro-myelinating factor that represses c-Jun activation. This slows down the cell reprogramming and, together with HDAC1, allows the synchronization of remyelination after axon regeneration [61]. To go further, DNA methylation [62] and Polycomb complex [52] have been shown to be involved in Wallerian demyelination, confirming that Schwann cell also undergoes epigenetic remodeling of the genome that leads to the spectacular fragmentation of the myelin sheath.

One path that Schwann cells follow when they demyelinate is probably the epithelial-to-mesenchymal transition (EMT) (Fig. 1), the process through which epithelial cells acquire migration and proliferation abilities. First, EMT factors Zeb2 was recently shown to be upregulated 6 h after nerve injury and when the gene was deleted Schwann cells were able to demyelinate but could not remyelinate [63, 64]. Zeb2 deletion did not affect c-Jun and Sox2 upregulation after nerve injury [63] but antagonized inhibitory effects of Notch and Sox2 on myelination [64]. This suggests that Zeb2 acts downstream of c-Jun and Sox2 and participates to remyelination by inhibiting dedifferentiation signals rather than activating demyelination process. Similarly, we found that Snai2, another gene characteristic of EMT, was upregulated after nerve injury in the sciatic nerve of mice (NT unpublished data). Second, EMT is characterized by a shift from E-cadherin expression in the epithelial phenotype to N-cadherin expression in the mesenchymal phenotype [65]. In peripheral nerves, E-cadherin is expressed in myelinating Schwann cells [36], while N-cadherin is expressed in pro-myelinating cells and precursors and re-expressed in demyelinating cells [66] (Fig. 1). Finally, the nuclear translocation of β-catenin in dedifferentiating Schwann cells following injury [18] may also represent an EMT-like process during demyelination. As myelinating Schwann cells show an epithelial-like polarization [67], these data suggest that demyelination is an EMT-like process where epithelial-like myelinated Schwann cell is reprogrammed to mesenchymal-like demyelinated cell.

## 1–4 h: mitochondria and MAPK pathways ignite and amplify the demyelination signal

Starting around 4 h after nerve injury, Schwann cell reprogramming, which follows c-Jun, Notch, and Sox2 activation, leads 20 h later to the first signs of myelin collapse and, 2 days later, to the complete disintegration of the myelin sheath. As Wallerian demyelination is triggered by axonal injury and degeneration, in order to reach the Schwann cell nucleus, axonal signal(s) have to be transduced into the glial cell and propagated in the cytoplasm. The role of MAPK pathways in this process is well documented.

Indeed, ERK1/2 signaling is robustly activated around 4 h after nerve injury [54, 68–70] and activation of the raf/MEK/ERK pathway is sufficient to induce demyelination of Schwann cells in vitro and in vivo [68, 71] (Fig. 1). Blocking this pathway partially prevented demyelination [71]; however, it did not prevent c-Jun activation [72, 73], suggesting that additional mechanisms are involved.

Indeed p38 is activated in Schwann cells as soon as 15 min after nerve injury in the lesion area and a bit later, 6 h, in more distal parts [74–77] (Fig. 1). Pharmacological inhibition of p38 activation or the enzyme deletion hindered demyelination, while its pharmacological activation in non-injured nerves was sufficient to induce both c-Jun phosphorylation and demyelination [76–78] ].

Another early event reported in Schwann cells after nerve injury is Rac1 activation and actin polymerization in Schmitt–Lanterman incisures [18]. While this actin polymerization is one of the first steps in the collapse of incisures occurring later during demyelination, it is not directly involved in c-Jun activation [18]. However, Rac1 has been shown to be essential for the activation of MKK7/JNK pathway in Schwann cells in culture suggesting this MAPK pathway may participate in c-Jun phosphorylation and demyelination [73] (Fig. 1). Indeed, while MKK7 activation promoted c-Jun phosphorylation in cultures [55] Rac1, MKK7 and JNK inhibition prevented it in nerve explant [73]. JNK expression and phosphorylation are quickly increased following injury in mouse sciatic nerves [55, 73] and ATF3, a transcription factor translocated in the nucleus after c-Jun activation via JNK, is also upregulated in Schwann cell nucleus [79]. However, as it has been reported that JNK activation is also a major event during axonal degeneration/regeneration after axotomy, it is possible therefore that JNK phosphorylation in injured nerves results from axonal injury and not from Schwann cell demyelination. ATF3 upregulation in Schwann cells nuclei may also result from c-Jun activation via p38 instead of JNK. So the role of MKK7/JNK pathway during Wallerian demyelination remains unclear.

The use of multiple MAPK pathways to induce the demyelination program in Schwann cells suggests that, beyond c-Jun and Sox2, numerous transcription and cotranscription factors are targeted during the process, underlining the concept of reprogramming (Fig. 1). In addition, it is very likely that multiple signaling pathways and basic cellular processes are affected by MAPK in demyelinating Schwann cells. Thus, Schwann cells physiology probably changes even before transcriptional modifications occur.

What is the origin of this massive and broad signaling resulting from the recruitment of MAPK pathways? Recently, we questioned the role of Schwann cell mitochondria during Wallerian degeneration. Using fluorescent probes targeted to mitochondria of Schwann cells in the crushed sciatic nerve of living mice [80], they found that mitochondria release a pulse of calcium in the cytoplasm just 1 h after crush. This mitochondrial calcium wave was necessary and sufficient to activate MAPK pathways and phosphorylate c-Jun thereafter (NT unpublished results). This indicates that mitochondria play an initial and central role during Schwann cell demyelination by releasing calcium that activates MAPK pathways (Fig. 1). In addition, this places mitochondria as the first target of an axonally initiated signal inside Schwann cells during Wallerian demyelination. Interestingly c-Jun upregulation that occurs in Schwann cells after crush was not prevented by blocking mitochondrial calcium release (NT unpublished results), showing that a mitochondria-independent pathway is likely to be also involved.

## 0–1 h: axonally derived signal is transduced in Schwann cells

Just like the very first moment of the Big Bang of the universe is crucial and unclear, the very first event(s) that triggers the Wallerian demyelination in Schwann cells is not known. There are many ways the Schwann cell can enter in the demyelination program but when a myelinated axon is severed, demyelination is triggered in less than 1 h in the distal part of the sciatic nerve and still the molecular triggers are not known. One hypothesis has nevertheless been raised.

In 2005, Guertin and collaborators [81] reported that as soon as 10 min after sciatic nerve cut, ErbB2/3 receptors were selectively phosphorylated in the distal clump of the nerve. Interestingly, this receptor activation did not last long—few hours—but a second long lasting activation appeared 3 days after the injury, suggesting that ErbB receptors were involved in two successive steps during Wallerian demyelination. Very early on, ErbB receptors phosphorylation occurred first in Schwann cell microvilli that cover the node of Ranvier and then propagated to the outer plasma membrane, abaxonal side, at the Schwann cell extremities [81] (Fig. 1). Pharmacologically inhibiting the receptors activation delayed Wallerian demyelination. As ErbB2/3 receptors is activated by Neuregulin 1, this suggested that this molecule, while required for Schwann cell survival, proliferation, and myelination [82], could also induce demyelination. Indeed it was also reported that Neuregulin 1-ErbB2 acted upstream of rac1-GTPase to regulate actin polymerization during Wallerian degeneration and inhibition of rac1 GTPase suppressed myelin fragmentation [18]. In addition, the aberrant activation of ErbB2 by *M. leprae* has been shown to lead to demyelination [83]. So a model of the initial Wallerian demyelination event includes the activation of ErbB2/3 receptors by Neuregulin 1 and the following amplification of the signal through Rac-MKK7-JNK and Ras-MEF-ERK1/2 pathways [68, 71, 73]. However, this model is not complete as genetic ablation of ErbB2 in adult Schwann cells did not produce a noticeable phenotype during Wallerian demyelination [84]. In addition, at the present time, there is no evidence that Neuregulin 1 or any other molecules that activates ErbB2/3 receptors is involved in triggering Wallerian demyelination. Thus, the signal that initiates Wallerian demyelination is still enigmatic and further studies addressing this issue are required not only for understanding the mechanism but also for providing therapeutic opportunities in the treatment of demyelinating diseases.

## Conclusion and perspectives

While the troubles resulting from the loss of myelin in peripheral nerves are serious and common enough in humans to define a specific class of peripheral neuropathy, limiting the term “demyelination” to the loss of myelin is strongly restrictive because they are many ways through which myelin can be lost. Among these different ways, Wallerian demyelination resulting from traumatic nerve injury is both the most common cause of demyelination and the simplest model of Schwann cell demyelination process. This model has been instrumental in the molecular and cellular characterization of the dedifferentiation process. More data and experiments are required to fully characterize Wallerian demyelination but a clear picture is already emerging. In order to consolidate it, more effort will probably be needed characterizing late visible “macroscopic” events such as ovoids formation and destruction. In addition other concomitants cellular events such as mitochondrial and metabolic changes and miRNA and small non coding RNA activity would probably add significantly to the global picture of Wallerian demyelination.

The most critical point is probably the earliest events that occur in Schwann cells when the axon initiates its self-destruction. Indeed, Wallerian demyelination appears to start in an amplification pathway that spreads from the extremities of the myelin to the nucleus of the Schwann cell. This molecular amplification allows the multiple changes that are required to dedifferentiate the cell. This scheme is characteristic of a program that always initiates from a code source. So if the final goal of scientists and medical doctors is to prevent pathological demyelination, then there is no use to try to target some factors involved in the program because alternative parallel events are probably already in course toward the deadly end. The only logical way would be to target the initial trigger of the program. Therefore, the earliest events are targets of choice for a therapeutic approach.

Finally, we have here limited our study to the Wallerian demyelination process, mostly reporting in vivo events following nerve crush or cut because in vitro or ex vivo demyelination models are prone to artifacts. Wallerian demyelination is not pathological because it is the natural response to the destruction of the axon. In the absence of demyelination axons do not regenerate and regrowth properly [1], so it is no use to prevent Wallerian demyelination. It is however worth noting that similar molecular and cellular events have been reported in many models of pathological peripheral nerve demyelination such as hereditary, toxic, metabolic, or autoimmune diseases. This suggests that while there are many ways through which demyelination can be triggered a similar or even identical program is engaged in all these demyelinating diseases. In these conditions, provided one can target the right earliest event of the program, it could be possible to block Schwann cell demyelination in the long term in multiple diseases with the same tool.

## Abbreviations

p75NTR: p75 neurotrophin receptor
NCAM: Neural cell adhesion molecule
GDNF: Glial cell-derived neurotrophic factor
BDNF: Brain-derived neurotrophic factor
Egr2: Early growth response protein 2
Mpz: Myelin protein zero
Mbp: Myelin basic protein
AP-1: Activator protein 1
NICD: Notch intracellular domain
Sox2: Sex determining region Y-box 2
MAPK: Mitogen-activated protein kinases
ERK: Extracellular signal-regulated kinases
MEK: MAPK-ERK-Kinase
MKK: Mitogen-activated protein kinase kinase
ATF3: Activating transcription factor 3
GTPase: Guanosine triphosphate hydrolase
